# Hierarchically embedded scales of movement shape the social networks of vampire bats

**DOI:** 10.1098/rspb.2023.2880

**Published:** 2024-04-24

**Authors:** C. Raven A. Hartman, Gerald S. Wilkinson, Imran Razik, Ian M. Hamilton, Elizabeth A. Hobson, Gerald G. Carter

**Affiliations:** ^1^ Department of Evolution, Ecology, and Organismal Biology, The Ohio State University, Columbus, OH 43210, USA; ^2^ Department of Mathematics, The Ohio State University, Columbus, OH 43210, USA; ^3^ Department of Biology, University of Maryland, College Park, MD 20742, USA; ^4^ Smithsonian Tropical Research Institute, Balboa, Ancón, Apartado Postal 0843-03092, Panama; ^5^ Department of Biological Sciences, University of Cincinnati, Cincinnati, OH 45221, USA

**Keywords:** fission–fusion, relationships, social networks, social structure, vampire bats

## Abstract

Social structure can emerge from *hierarchically embedded scales of movement*, where movement at one scale is constrained within a larger scale (e.g. among branches, trees, forests). In most studies of animal social networks, some scales of movement are not observed, and the relative importance of the observed scales of movement is unclear. Here, we asked: how does individual variation in movement, at multiple nested spatial scales, influence each individual's social connectedness? Using existing data from common vampire bats (*Desmodus rotundus*), we created an agent-based model of how three nested scales of movement—among roosts, clusters and grooming partners—each influence a bat's grooming network centrality. In each of 10 simulations, virtual bats lacking social and spatial preferences moved at each scale at empirically derived rates that were either fixed or individually variable and either independent or correlated across scales. We found that numbers of partners groomed per bat were driven more by within-roost movements than by roost switching, highlighting that co-roosting networks do not fully capture bat social structure. Simulations revealed how individual variation in movement at nested spatial scales can cause false discovery and misidentification of preferred social relationships. Our model provides several insights into how nonsocial factors shape social networks.

## 1. Introduction

Social networks are a useful tool with applications across many ecological and evolutionary contexts, including pathogen transmission, information transmission, dominance, social integration, and many more [1–6]. To properly interpret social networks, it is important to study the underlying mechanisms that shape the observed network structure. In most animal social networks, connections are defined by associations (co-occurrences in space and time), and these networks are therefore directly shaped by how individuals move relative to each other. However, the motivations for these movements cannot be directly measured using observational data.

Movements of individuals and their resulting associations are influenced both by social preferences (e.g. attraction or repulsion) and by multiple nonsocial factors such as spatial preferences and constraints on movement from health, energetics, or habitat structure [7,8].

Individual variation in movement can therefore cause individual variation in social connectedness—even in the absence of variation in social preferences. All else being equal, an individual that has a larger home range, or one that moves more frequently within the same area, is expected to have more social encounters than a stationary individual. In studies with house mice (*Mus musculus*) and common vampire bats (*Desmodus rotundus*), experimentally induced lethargy reduced the social connectedness of random individuals relative to control individuals [9–11]. A causal relationship between movement rate and social connectedness can exist independently of variation in social traits or preferences.

Movement rates vary across individuals, but they are also explained by external nonsocial factors such as food distribution, habitat structure or roost architecture [12–14]. Consequently, social structure often emerges from *hierarchically embedded scales of movement*. For example, long-tailed tits (*Aegithalos caudatus*) can switch between flocks [15], within each flock they can switch their roosting site [16], and within each roosting site they can switch their interactions among different flock mates. At each single scale, such movement patterns are called ‘fission–fusion dynamics', because groups of individuals repeatedly divide into smaller subgroups that vary in composition and size over time, and then re-aggregate into larger groups [17]. However, when fission–fusion dynamics occur on multiple spatial scales simultaneously, each scale of social dynamics is influenced by other larger scales.

These hierarchically embedded scales of movement occur in many kinds of animal societies. In more ‘fluid’ fission–fusion societies, individuals move between temporary groups [17] and switch interaction partners within each group. In more structured ‘multi-level societies', individuals form relatively stable, core sub-groups that fuse with other groups [18], and individuals can move among interaction partners within and among sub-groups. Even highly stable societies can be shaped in subtle ways by hierarchically embedded scales of movement. For example, the social organization of eusocial paper wasps (*Polistes canadensis*) can be described by three spatial scales: nests, aggregations and communities [19]. Workers move among cells at their home nest to feed different larvae, but as the need for additional feeding decreases, they will sometimes move to neighbouring nests (inter-nest drifting) to feed larvae that are less related but more in need [20]. Such hierarchically embedded scales of movement create social dynamics that can be modelled as *hierarchically embedded networks* [21], where connections at one scale constrain and affect connections at different scales, or as *multi-layer networks* [22], where different layers represent associations at different scales.

**Figure 1.**
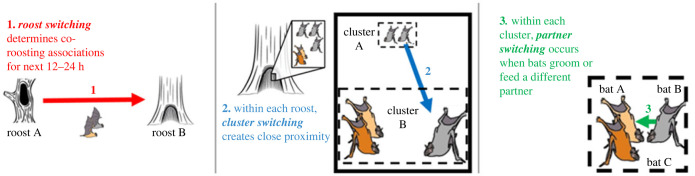
Three spatial scales of movement of vampire bats. Bats move between roosts from day to day (red arrow 1). Additionally, inside each roost, bats aggregate into clusters of touching bats and can move between those clusters (blue arrow 2) multiple times as the day progresses. Inside each cluster, bats move towards a partner (green arrow 3) to groom them or share food.

Common vampire bats are a clear example of a species where hierarchically embedded movements shape social behaviour. In this species, social grooming networks are a useful measure of preferred social relationships because they are directed investments of time and energy, and juveniles and adult females use grooming to build food-sharing relationships [23–26]. However, the grooming partners available to a vampire bat at any point in time are determined by at least three hierarchically embedded scales of movement: (1) *roost switching*, (2) *cluster switching* within roosts, and (3) *partner switching* within clusters (figure 1).

Roost switching has been studied in Costa Rica, where vampire bats roosted in hollow trees each day but switched among a set of multiple trees from day to day [27], similar to many other forest-dwelling bat species [28–31]. These roost-switching movements determine co-roosting associations. The resulting *co-roosting networks* based on visual observations or passive integrated transponder (PIT) tags are the most common way to describe the social network structure of bats [32–35]. Within each roost, cluster switching occurs when vampire bats move between discrete clusters of bats in close contact. The resulting *clustering networks* can be created from visual observations or proximity loggers [36,37]. Within a cluster, partner switching occurs when bats switch their partner by directing their grooming to different bats in their cluster. Each scale of association from roost to cluster to partner constrains the possible partners at smaller embedded scales. The duration of these constraints also varies at each scale. For example, each roosting cluster constrains the partners that a bat can groom at that moment, but the choice of roost tree at sunrise constrains the set of available roost mates until the next sunset, about 12 h later [38].

There are several aspects of this nested social structure that remain unclear. First, it is unclear how individual variation in movement rates at these different scales influences overall social connectedness. Second, the relationships between these three movement types (roost, cluster and partner switching) are also unclear. They may be positively correlated if a behavioural syndrome affects movement across all scales. Additionally, they may be independent if there are different reasons for each type of movement. Finally, it is unclear to what extent individual variation in movement at hierarchically embedded scales complicates the identification and measurement of preferred social relationships.

Here, we ask: to what extent does an individual vampire bat's movement rate at three hierarchically embedded spatial scales predict the number of partners groomed (i.e. grooming outdegree centrality)? To answer this question, we first used existing empirical data to test (1) if observed grooming outdegree is predicted by roost switching in wild vampire bats [27,39] and (2) if grooming outdegree is predicted by rates of cluster switching or partner switching in captive vampire bats [40]. However, these two empirical analyses cannot disentangle how a bat's social connectedness is influenced by its general propensity to move versus its attraction to certain partners or spaces. Therefore, to understand the *causal* relationships between movement and grooming network centrality in the absence of social and spatial preferences, we created an agent-based model.

Agent-based models are an excellent way to study emergent properties resulting from simple, individual-level decision rules or traits. In the past, these models have often been used to study movement personality [41], as well as movement at multiple scales [42–44]. However, to our knowledge, no agent-based model has been used to quantify the relationship between social connectedness and individual variation in movement rates at hierarchically embedded scales.

## 2. Methods

### (a) Empirical analyses

We analysed existing published data to estimate how often common vampire bats switched roosts, clusters and partners. To estimate individual rates of roost switching, we used 1336 observations of 81 free-ranging bats of both sexes (38 males and 43 females) that were observed more than 25 times across 11 tree roosts along the Rio Corobici in Guanacaste, Costa Rica [27,39]. We also made grooming networks using 1761 grooming interactions among 27 of these bats [23]. To estimate individual rates of cluster switching and partner switching, we used 4092 observations of clusters (defined as bats roosting in the same corner of a flight cage) and 22 836 observations of grooming from 31 vampire bats of both sexes (5 males and 26 females) at a captive colony in Panama [40]. Individuals in both studies were identified visually using unique combinations of distinctive wing bands.

To estimate roost-switching rates, we only used observations of the same bat or roost on consecutive days, because roost switching would be underestimated when a bat moved away and then returned to the same roost between observations (see electronic supplementary material for details). To calculate cluster-switching and partner-switching rates, we counted consecutive observations of the same bat where a switch occurred, then divided that count by the total time elapsed between those observations (see the electronic supplementary material for details). We only considered consecutive cluster-switching and partner-switching observations that occurred within a sampled hour. To calculate *within-cluster* partner-switching rates, we did not count partner switches and the associated time lapse that occurred owing to partner switches between clusters.

To create co-roosting and co-clustering networks, we defined edge weights in the co-roosting and co-clustering networks as the ‘simple ratio index’ of association [45–47]. To create grooming networks, we defined edge weights as total minutes of grooming. To assess within-bat correlations between movement types, we used a linear model to test if cluster-switching rates predicted within-cluster partner-switching rates.

To determine how well roost-, cluster- and partner-switching rates predict the overdispersed counts of the number of bats groomed (outdegree centrality), we fitted a quasi-Poisson generalized linear mixed-effects model with each of the three rates as single predictors, and bat as random intercept. We used nonparametric bootstrapping to create a 95% confidence interval (CI) around the standardized coefficient (*b*).

### (b) Agent-based model

We created a model using NetLogo 6.2.0 and used it to simulate movements of virtual vampire bats that lacked preferences for roosts, clusters or partners. Each of 11 roosts contained four locations for potential clusters. We randomly assigned each virtual bat to a starting roost and cluster location. For each spatial scale, each bat had a switching propensity randomly sampled with replacement from empirical estimates of the probabilities of movement. Switching probabilities at every scale were conditional on the time since the last switch (see electronic supplementary material).

We initially ran all the simulations with populations of 200 virtual bats, the approximate number of bats encountered and banded by Wilkinson along the Rio Corobici between 1978 and 1983 [27,39]. To explore how our results would change with fewer bats and limited partner choice, we later ran the simulation with 100 virtual bats to explore how our results would change with fewer bats, leading to smaller group sizes and limited partner choice (2.3 bats per cluster, or an average of 1.3 partners per cluster).

To isolate the effects of movement, we fixed the probability of grooming per minute for all virtual bats at 1.8% (the mean probability that a captive vampire bat groomed another bat during the sampled hours from empirical observations of captive vampire bats [40]). We included a synchronous 200 min foraging period when bats left all roosts to forage outside the roosts. The simulations recorded observations of behaviours every minute for 15 days.

When in a roost, virtual bats randomly decided every minute whether to groom a partner and whether they would switch partners based on an increasing probability related to the time since last switch at that scale. The decision was solely determined by the groomer initiating the exchange; the receiver did not decide whether to accept grooming. Each bat could only groom one partner in any particular minute, but multiple bats could groom the same bat during that minute. Virtual bats decided whether to switch clusters within their roost once every hour. Additionally, they decided whether to switch roosts once per day after returning from foraging.

If a bat changed its partner as a result of cluster or roost switching, we did not count this event as partner switching. Similarly, if a bat changed clusters owing to roost switching, we did not count this event as cluster switching. We took this approach to test the effects of a bat's decisions at each scale rather than the effect of what it experiences. Although we measured within-roost cluster switching and within-cluster partner switching, for brevity these are simply referred to as ‘cluster switching’ and ‘partner switching’.

### (c) Simulations using agent-based model

We ran five types of simulation, each 100 times, and we ran those five simulation types across two different population sizes, once for 100 bats and again for 200 bats. Each of the five simulation types had switching propensities that were either fixed or individually variable and either correlated or uncorrelated. In simulation 1, virtual bats were assigned a random propensity of roost, cluster and partner switching; these propensities were uncorrelated within each individual bat because they were drawn independently from empirical distributions. The resulting standardized coefficients of the switching rates from this simulation measured how well each movement type predicted grooming outdegree when controlling for the other movement types.

In simulations 2–4, one type of movement varied among bats while the two others were fixed (to the mean observed from the empirical data). In simulation 2, only roost-switching propensity varied across individuals. In simulation 3, only cluster-switching propensity varied across individuals. In simulation 4, only partner-switching propensity varied across individuals.

Using simulations 2–4, we estimated the *reference effects*, defined as the median standardized coefficients of the switching rate when switching propensity was *not* variable between bats. The *reference effects* measure how well one movement type predicts grooming outdegree when it lacks individual variation in switching propensity. We estimated the *isolated effects* of individual variation in each switching propensity, defined as the difference between the standardized coefficient of the switching rate when only it was variable between bats and the reference effect. The *isolated effect* measures how well individual variation in only one movement propensity predicts grooming outdegree while accounting for the reference effect.

Simulation 5 was similar to simulation 1 except that the three switching propensities were positively correlated, such that virtual bats that moved most frequently at one scale also moved most frequently at other scales (see electronic supplementary material). By comparing the results of simulations 1 and 5, we could therefore assess the effect of switching propensities being correlated (simulation 5) or uncorrelated (simulation 1).

In sum, our model allowed us to ‘switch on and off’ the existence of realistic individual variation in movement at each spatial scale to isolate the social consequences for the individuals, while eliminating the confounding effects of social and spatial preferences found in real vampire bats. By adding or removing individual variation in movement at each scale or across all scales, and by making these movements correlated or not across scales, these simulations allowed us to isolate the causal effects of individually variable movement on grooming network centrality.

Note that a bat's assigned probability of switching (its switching *propensity*) is not the same as the number of times it actually switched during the simulation (its switching *rate*). When switching propensity was fixed, all bats with the same time since last switch also had the same probability of switching at that time step. However, as the model was randomized, the realized number of switching events differed, even when bats had the same switching propensity. This can be seen in equations (2.1) and (2.2), where *o* is the odds of a switch at that time step, *p* is the probability of a switch, *a* is the intercept of a logistic mixed effects model (which could be set to be variable or equal for all bats), *b* is the slope of the logistic mixed effects model (which was always the same across bats), and *t* is the number of time steps since last switch. When a particular switching propensity was fixed, *a* was the same for all bats (and, consequently, *p* if *t* was also the same). However, every time step, the virtual bats generated an independent value, *r*, that, if less than *p*, signalled the bat should try to switch partners. Because each bat generated a different *r* every time step, the realized partner switching rate was different by bat:

2.1 ln⁡o=a+bt

and

2.2 $$p=\frac{o}{1+o}.$$

The goal of these simulations was to manipulate the switching *propensity* (which cannot be measured from the empirical data) and then assess the relative effect of the resulting individual differences in observed switching *rate*. For a more detailed description of the agent-based model, see Overview, design concepts, details (ODD) in the electronic supplementary material.

### (d) Analysis of simulated data

Over the course of 15 simulated days, we counted cases of roost, cluster and partner switching. We used grooming rates to create the grooming network. We assessed two measures of grooming network centrality: *outdegree*, the number of grooming recipients groomed by the focal bat, and *pagerank*, which estimates connectedness using both direct ties (grooming receivers) and indirect ties (grooming receivers of those receivers). To estimate pagerank based on grooming given rather than grooming received, we calculated it from the transposed grooming matrix. To assess how each movement type predicted grooming network centrality, we fit a Poisson generalized linear model with outdegree (count of partners groomed) as the response, and the scaled counts of roost-, cluster- and partner-switching events as predictors. We did not detect evidence for over-dispersion. Effects on outdegree and pagerank centrality were almost identical (see electronic supplementary material for results), so we focus our discussion on outdegree.

### (e) Effect of individual variation in movement and habitat structure on tests for preferred relationships

Individually variable movements and hierarchically embedded habitat structures create highly nonrandom association rates that could be taken as false evidence of social differentiation (preferred social relationships) if these features are not properly controlled for in the analysis [8]. Type 1 error means that social preferences might be falsely detected, exaggerated or inaccurate when inferences ignore the role of habitat structure and individual variation in movement. To explore this, we used permutation tests to test the significance of social differentiation, the coefficient of variation of edge weights, which is a standard method to test for social preference [45,48]. We did 50 permutation tests for detecting social differentiation using 5 randomly selected grooming networks for each of the 10 simulations. To calculate the *p*-value, permutation tests were repeated 100 times, each with 10 000 permutations.

To illustrate the importance of constraining permutations to account for habitat structure, we first used *unconstrained permutation tests*, which permuted the partners across all observed events from randomly selected simulations. Next, to investigate the effects of spatial and temporal constraints on permutations, we used two types of constrained permutation tests on the same simulation. In the *semi-constrained permutation test*, we created the null model by only permuting groomed partners observed on the same day and in the same roost. In the *highly constrained permutation test*, we created the null model by only permuting partners observed in the same hour and cluster. Because virtual bats had no social preferences, any inferences of preferred relationships constituted type 1 error. We ran each of the demonstrative permutation tests 100 times, each with 100 000 permutations to the partner groomed.

Next, we tested which factors contributed to false appearance of preferred relationships. To do this, we used unconstrained permutation tests to check for social differentiation in data generated by four additional ‘control simulations’, each with less complexity. The first control simulation lacked hierarchically embedded scales of movement, because all virtual bats were in a single cluster. The second control simulation lacked both hierarchically embedded scales of movement and individual differences in partner-switching propensity. Although partner-switching propensity was constant across all bats, each bat still had a greater probability of grooming the same partner in series rather than grooming a new partner, which we call ‘byproduct partner fidelity’. The third control simulation was the same as the second but it removed byproduct partner fidelity: instead of a bat deciding whether or not to switch partners, it selected a random available partner. Finally, the fourth control simulation was identical to the third except all bats groomed simultaneously. We used these control simulations to establish which created the false appearance of social differentiation. Again, we ran these latter demonstrative permutation tests 100 times, each with 100 000 permutations.

## 3. Results

### (a) Empirical results

**Figure 2.**
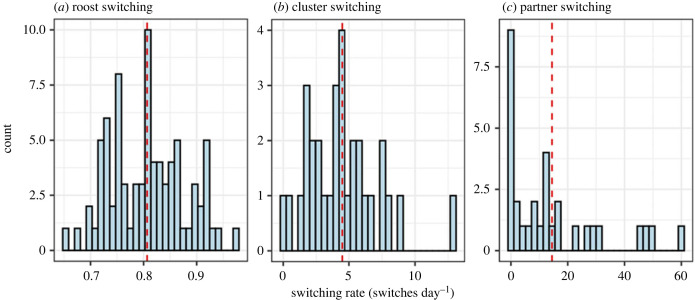
Distribution of empirical switching-rate estimates across bats. (*a*) The number of roost switches per day, estimated using only roost or roost association observations within a day of each other. (*b*) The number of cluster switches per day, estimated using only association observations within an hour of each other. (*c*) The number of partner switches per day, using only grooming observations within an hour of each other. The mean switching rate is represented by the red line.

We estimated that the 81 wild vampire bats switched roosts on average once every 1.25 days, or 0.81 (range: 0.65, 0.97) switches per day, and that the 31 captive vampire bats switched clusters (cage corners) 4.46 (range: 0.00, 12.88) times per day. Partner switching in the same group of captive vampire bats occurred on average 14.46 (range: 0.00, 59.96) times per day (figure 2). Captive bats that more frequently switched clusters did not more frequently switch grooming recipients, i.e. cluster-switching rate did not predict within-cluster partner-switching rate (*R*^2^ = 0.004, *b* = 0.06, 95% CI: −0.22–0.35, *n* = 29, *p* = 0.74; electronic supplementary material, figure S1).

**Figure 3.**
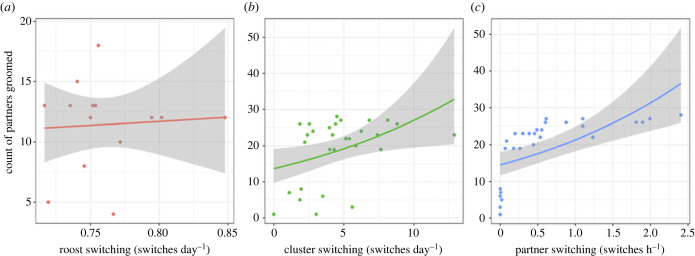
Empirical data show that movements within roosts predict the number of partners groomed. Number of partners groomed is shown by individual rates of roost switching (left), cluster switching (middle), and within-cluster partner switching (right). Shaded intervals are 95% CI on the slope. The relationship between outdegree and cluster- and partner-switching rate appears nonlinear.

We did not detect a clear relationship between the number of partners groomed (outdegree centrality) and roost-switching rates (*b* = 0.02, 95% CI: −0.09–0.16, *n* = 15, *p* = 0.80, figure 3*a*). However, outdegree was predicted by rates of within-roost cluster switching (*b* = 0.23, 95% CI: 0.07–0.49, *n* = 29, *p* < 0.001, figure 3*b*), and within-cluster partner switching (*b* = 0.30, 95% CI: 0.18–0.43, *n* = 29, *p* < 0.0001, figure 3*c*).

### (b) Agent-based simulation results

**Figure 4.**
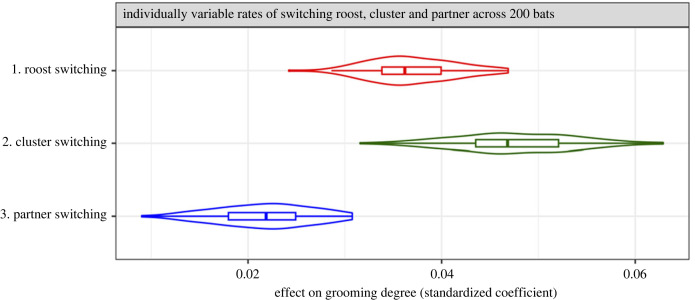
Effects on grooming outdegree centrality when individual variation in movement propensity is independent across scales. Panels show results when there are 200 simulated bats. Violin plots and box plots show the distribution of effects (standardized coefficients) of roost switching, cluster switching and partner switching on grooming outdegree for each type of simulation.

In simulation 1, when the switching propensities at all three scales varied among bats simultaneously and independently, cluster-switching rate was on average the strongest predictor of grooming outdegree, both with 200 virtual bats (figure 4; electronic supplementary material, figure S2*a*) and with 100 virtual bats in the simulation (electronic supplementary material, figure S3*a*). Decreasing the population of virtual bats (and the density of bats to 2.3 bats per cluster) in the simulation increased the effect of partner switching on the number of partners groomed. However, there was no significant difference between population sizes in the effect of roost switching or cluster switching on grooming outdegree (compare electronic supplementary material, figure S2*a* with figure S3*a*). Results were similar when switching rates across scales were correlated (electronic supplementary material, figures S2 and S3*e*), but the effect of cluster switching was reduced.

**Figure 5.**
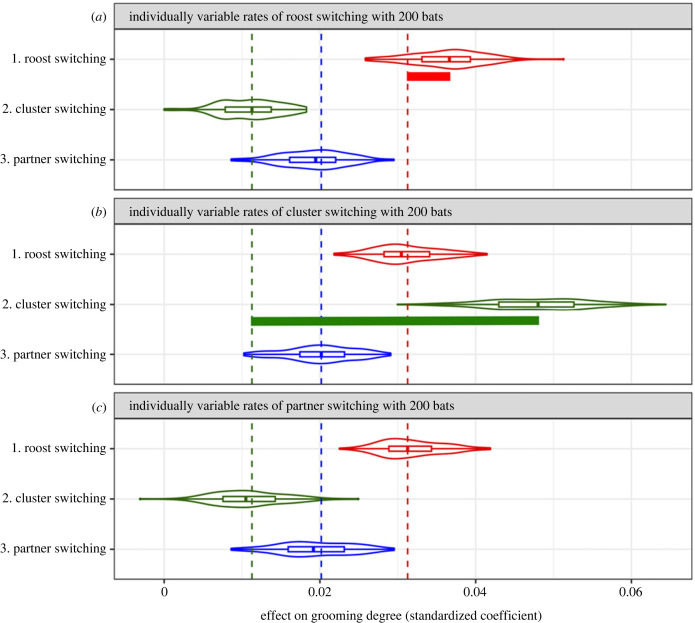
Observed effects versus reference effects with 200 bats. Plots show predictors of grooming outdegree centrality when one type of movement rate is individually variable while the other two types of movement lack individual variation in movement propensity. Plots show results when only probabilities of roost switching varied (*a*), when only probabilities of cluster switching varied (*b*), and when only probabilities of partner switching varied (*c*). Coloured dashed lines show the greater of the two median reference effects, or the effects when there is no variation in movement at that scale. For example, the red reference effect line passes through the median effect in plot (*c*). Coloured bars show the isolated effect when applicable, or the positive distance from the reference effect, resulting from individual variation in movement.

We expected when virtual bats had consistent individual variation in movement propensity at only one scale, that scale of movement would be the strongest predictor of grooming network centrality (outdegree and pagerank). This prediction was confirmed in four cases, but it was surprisingly not met in two cases. First, when only roost-switching propensity varied among 100 virtual bats, it was partner switching that was the best predictor of centrality (electronic supplementary material, figure S3*b*). Second, when only partner-switching propensity varied among 200 virtual bats, roost switching was the best predictor of centrality (figure 5*c*; electronic supplementary material, figure S2*d*). We explain this unintuitive result in the Discussion under ‘Insight 1’.

The *reference effect* on grooming centrality measures the effect of each movement type when bats did not vary in their propensity to move at that scale (marked by the dashed lines in figure 5). Note that reference effects were consistently above zero even though there was no individual variation in switching propensity at that scale (figure 5; electronic supplementary material, figure S3*b–d*), because movement events were probabilistic and grooming outdegree was predicted by random variation in movement within the spatial and temporal constraints. One could consider the reference effect to be the effect of hierarchically embedded scales of movement determined by the habitat structure and time constraints. With 200 bats, roost switching had the strongest reference effect, followed by partner and cluster switching (figure 5; electronic supplementary material, figure S2). With 100 bats, the roost-switching and cluster-switching reference effects were similar to when there were 200 bats, but the partner-switching reference effect was greater than the roost-switching reference effect (electronic supplementary material, figure S3).

The *isolated effect* on grooming centrality is the effect of individual variation in movement propensity (marked by the horizontal bars in figure 5), and it is defined as the positive distance between the reference effect (in the absence of individual variation) and the median standardized effect of that switching rate on centrality in the presence of individual variation. The isolated effects describe the impact of adding individual variation in movement propensity, or personality, at each scale. The isolated effect of cluster switching was much greater than the isolated effect of roost switching, and individual variation in partner-switching propensity did not appear to significantly increase the effect of partner switching on centrality (figure 5*c*; electronic supplementary material, figures S3*d* and S4*d*).

When cluster-switching propensity varied across bats, cluster-switching rate had a greater effect on outdegree than did its reference effect in 99–100% of cases (electronic supplementary material, table S1). Similarly, when roost-switching propensity varied across bats, roost-switching rate had a greater effect on outdegree than did its reference effect in 65–92% of cases. However, when partner-switching rate varied across bats, it did not usually have a greater effect on outdegree than did its reference effect; the observed effect was greater than the reference effect in only 36–65% of cases (mean = 45.5%). As previously mentioned, these results for the effects of switching on outdegree were almost identical for pagerank, varying by 0–4% (for effects of movement on pagerank, see electronic supplementary material, figures S4 and S5).

### (c) Effect of individual variation in movement and habitat structure on tests for preferred relationships

*Unconstrained permutation tests* incorrectly detected social differentiation (preferred social relationships) in all grooming simulated networks (*p* < 0.01 in all 50 tests run). When we removed hierarchically embedded scales of movement and individual variation in partner-switching propensity, type 1 error persisted but the gap between expected and observed social differentiation decreased (electronic supplementary material, figure S6). Unconstrained permutation tests correctly failed to detect social differentiation only in simulations where we removed both hierarchically embedded scales of movement and the byproduct partner fidelity resulting from a propensity to stay with the last groomed bat (electronic supplementary material, figure S6).

Permutation tests for evidence of preferred relationships could not control for hierarchically embedded scales of movement and byproduct partner fidelity, even when using the most constrained data permutations (electronic supplementary material, figure S7). The difference between the observed and expected social differentiation was reduced in *semi-constrained permutation tests* that permuted data within day and roost, but this test still incorrectly detected social differentiation in all simulated networks (electronic supplementary material, figure S7*b*). Even in the *highly constrained permutation tests*, we incorrectly detected social differentiation, despite further reduction in the difference between observed and expected (electronic supplementary material, figure S7*c*). We explain these results in the Discussion under ‘Insight 5’.

## 4. Discussion

Our goal was to understand the causal effects of individual variation in movement at multiple nested spatial scales on network connectedness. To do this, we used agent-based simulations, parameterized with empirical data from vampire bats. We first estimated roost-switching rates from field observations and estimated cluster-switching and partner-switching rates from captive observations. We then used the distributions of these movement rates to make an agent-based model that simulated virtual bats moving at all three scales simultaneously, without social and spatial preferences. By eliminating social and spatial preferences, we isolated and tested the network consequences of hierarchically embedded scales of movement. By adding and removing individual variation in switching propensity at each scale of movement, we tested the effect of movement propensity (or movement ‘personality’) on a bat's social connectedness. By adding and removing within-bat correlations between movement types, we tested the effect of a movement-based behavioural syndrome. Through these manipulations, we generated five key insights.

### (a) Insight 1. Individual variation in movement propensity at multiple spatial scales influences social connectedness in complex nonintuitive ways

Analyses of empirical data were consistent with the expectation that movement rates predict social connectedness. Although we failed to detect a clear relationship between grooming outdegree (number of partners groomed) and roost-switching rates in 15 wild vampire bats, we did find that grooming outdegree was predicted by both cluster switching and within-cluster partner switching in 29 captive vampire bats. A statistical relationship between movement and outdegree centrality is consistent with two non-mutually exclusive causal effects: (1) social motivations causing more movement and (2) movement causing more social interactions. To study the latter causal effect, we used agent-based simulations with virtual bats, which lacked social and spatial preferences.

The simulations show how variation in network centrality is impacted both by individual variation in movement propensity and by hierarchically embedded scales of movement. Although the exact effects of movement at any scale cannot be extrapolated to real vampire bats, which do have social preferences, one can interpret the relative importance of each scale of nonsocial movement on network centrality. Overall, we found that a greater propensity for roost switching and cluster switching increased a bat's grooming centrality. In contrast to real vampire bats, however, individual variation in within-cluster partner-switching propensity was not a clear predictor of grooming outdegree.

The simulations allowed us to disentangle the effects of individual movement from the effects of the fixed habitat structure. For instance, we found that in the absence of consistent individual variation in each switching propensity, grooming outdegree was influenced more by random variation in roost-switching rates than by random variation in cluster-switching rates. However, adding individual variation in cluster switching had a greater impact on network centrality than adding individual variation in roost switching. Put differently, within-roost movement ‘personality’ influenced social connectedness more than between-roost movement ‘personality’.

Some results were highly nonintuitive. For example, we predicted that adding consistent individual variation to only one type of movement would cause it to be the best predictor of centrality, but this prediction was not met in two of six cases. In one case, adding individual variation to roost switching increased its effect beyond its reference effect, but not beyond the reference effect of partner switching (electronic supplementary material, figure S3*b*). Such patterns can occur because the observed switching rate is constrained by other factors besides the individual's actual switching propensity. In another case, after adding consistent among-individual variation in partner switching, the observed effect did not even increase above its reference effect (figure 5*c*; electronic supplementary material, figure S2*d*). One reason that adding individual variation to partner switching had no effect was that the observed partner-switching rates were restricted by whether there was a new partner available to groom in the same cluster. Another reason is that partner switching was the only movement type that required grooming. If grooming was more frequent, the effect of individual variation in partner switching propensity would likely be greater.

Taken together, these findings highlight the emergent complexity of social systems with hierarchically embedded scales of movement. They also emphasize the advantage of identifying non-zero reference (null) effects through simulation [8,49]. To model a hypothesized data-generating process, it is useful to simulate the effect sizes expected from random behaviours to identify other causes of network structure beyond the effect of interest.

### (b) Insight 2. Co-roosting networks in bats might not fully capture social structure

When hierarchically embedded scales of movement exist, variation in movement at larger scales dictates the availability of potential partners at smaller scales, but movement at smaller scales determines what proportion of those potential connections are realized. Based on our simulations, which found that cluster-switching rate was more important for influencing outdegree centrality than roost-switching rate (figure 3; electronic supplementary material, figure S4*a*), we suggest that social network dynamics will be shaped more by movements at smaller scales when individuals (1) vary in movement at the smaller scale, (2) have many possible interaction partners at the smaller scale, and (3) can move at the smaller scale more often than at a larger scale. These conditions are met for vampire bats, many other bat species, and several other species with fission–fusion dynamics.

In bats, most social networks are based on co-roosting networks shaped by movements between roosts [27,32–35,39,50–52]. Both empirical analyses and data-driven simulations suggest that within-roost movements are also important, and probably more important than between-roost movements, for determining a vampire bat's grooming network centrality. The greater effect of within-roost movements over between-roost movements is caused by greater individual variation in within-roost movements and by more opportunities to move within roosts than between roosts. Although frequent roost switching provided access to more potential partners, within-roost movements determined the proportion of those potential new partners that were groomed. The importance of within-roost movement will also depend on roost architecture. Some roosts (e.g. crevices in bridges) permit little movement, whereas others (e.g. large caves) allow many possible configurations of roosting bats. It would therefore be interesting to study how roost architecture impacts social structure in bats.

The general principle that either between-roost or within-roost dynamics do not fully capture social structure is applicable to many other species that use multiple roosting sites or foraging sites whose composition can change day to day. This is true for any study where observation occurs at a spatial or temporal scale that is too coarse to capture important social interactions, such as when observation of movements at smaller scales is limited. Outside bats, other examples of cases where small-scale interactions may be hidden include field studies that track animals that aggregate in large groups using low-resolution GPS, animals that have small-scale interactions underwater, or burrowing animals that interact belowground [53].

### (c) Insight 3. Partner switching is shaped by interactions between habitat structure and population size

The effect of partner switching on grooming outdegree differed between simulations with 200 (mean network density = 0.48, range: 0.47, 0.49) versus 100 bats (mean network density = 0.55, range: 0.53, 0.57), because population size determined the number of possible partners in each roost and cluster. In simulations with 100 bats, partner switching was often not possible, because each cluster had on average only 2.3 bats. As such, when a new potential partner did enter a cluster, that partner would likely be groomed, resulting in a greater proportion of roost mates being groomed. With 200 bats, the average cluster size was 4.5 bats, meaning that each bat is more likely to groom the same set of bats, with no greater likelihood to interact with new partners than bats that were already sharing a cluster. As partner switching had a greater effect on the number of partners groomed in smaller populations, these simulations had greater network density. Although movement rates influence social dynamics, so too do the number of individuals and the number of roosts and clusters. Differing numbers of roosts or clusters would change the relative effects of roost and cluster switching by providing more or fewer opportunities to interact with different proportions of the population. This model illustrates how habitat structure can impact possibilities for partner choice and switching, which can also have a major influence on cooperative behaviour [54].

### (d) Insight 4. Behavioural syndromes might have subtle impacts on the relative importance of different hierarchically embedded scales of movement

Behavioural syndromes are correlations among repeatable behaviours, including individual variation in movement [55]. For instance, individual that we might label as ‘high-movement bats’ might be more likely to switch roosts, clusters and partners. Simulation 5 (correlated movement among variable switching propensities) showed that effects of movement types on centrality were similar whether correlated or uncorrelated; however, correlated movement rates did seem to reduce the effect of cluster switching (electronic supplementary material, figure S4*a* versus S4e and S5a versus S5e), suggesting that correlations between movement types (a behavioural syndrome) could impact which movement type has the largest effect on network centrality. In real vampire bats, we did not detect evidence for a behavioural syndrome linking rates of cluster switching and within-cluster partner switching, but we had limited statistical power and we could not compare within-roost with between-roost movement variation. A more accurate assessment of these correlations would require tracking movement and interactions at all scales with higher sampling effort.

### (e) Insight 5. Hierarchically embedded scales of movement distort identification and measurement of social preferences

A common analysis in animal social network analysis is testing for preferred relationships, or social differentiation. The null hypothesis is that the standardized variation in association rates (network edge weights) matches what one expects from randomized associations. One challenge of these tests is that their proper use requires controlling for nonsocial drivers of network structure such as sampling biases, habitat structure and temporal effects through the use of constrained data permutations [8]. We used our agent-based model to explore this challenge because the virtual bats lacked social preferences, but their association rates were influenced by hierarchically embedded scales of movement. We tested the effectiveness of several permutation-based null models when ignoring or accounting for hierarchically embedded scales of movement. To do this, we first generated datasets from the agent-based simulation. We then tested for social differentiation using three permutation tests with null models of increasing complexity: unconstrained, semi-constrained and highly constrained (electronic supplementary material, figure S7). The unconstrained and semi-constrained permutation tests incorrectly reported social differentiation because they did not fully account for the hierarchically embedded scales of movement. The highly constrained permutation test which permuted possible events within each hour and cluster also incorrectly detected preferred relationships (electronic supplementary material, figure S7*c*), which we attribute to a tendency to continuously interact with the same individual (byproduct partner fidelity), which was lacking in our null model.

To understand why hierarchically embedded scales of movement created biased estimates of social preference, we again applied the unconstrained permutation test to our simulated data, but then reduced the complexity of the generative agent-based model until false evidence for social differentiation disappeared (electronic supplementary material, figure S6). This procedure demonstrated that false social differentiation resulted from two processes: hierarchically embedded scales of movement and byproduct partner fidelity that emerges when bats are more likely to stay with the same partner for extended periods of time. Although real vampire bats do have social preferences [56,57], these results show that habitat structure and nonsocial aspects of personality that create individually variable movement rates might create, reverse or distort the evidence for these social preferences.

The importance of byproduct partner fidelity in creating nonrandom social structure (electronic supplementary material, figure S6*d*) and the failure to correctly identify a lack of social preference, even in constrained permutation tests (electronic supplementary material, figure S7*c*), illustrate how permutation tests may be incapable of correctly identifying social differentiation if individuals tend to associate with the same partner repeatedly for nonsocial reasons. Byproduct partner fidelity might be a realistic feature of animal behaviour, because an animal surrounded by four individuals will find it slightly easier to groom the partner it is currently facing rather than grooming the individual behind it. In permutation approaches that permute events to different individuals, the animal is assumed to be equally likely to groom any of the four individuals regardless of what happened in the previous time step. To account for this, permutations must control for the switching rate, by only swapping recipients of partner switching rather than swapping recipients of partner grooming. Generative models, such as agent-based simulations, can be used to test permutation approaches and discover their limitations [8].

These results illustrate the difficulties of disentangling social preferences from nonsocial drivers of nonrandom social network structure, and suggest that agent-based simulations can provide a useful tool, not only for understanding empirical data [49], but also for testing different methods of hypothesis-testing with network data [8,49]. For example, a promising future direction in network analysis is using Bayesian inference to model social networks [58,59], including in ways that maintain uncertainty in edge weights for subsequent statistical inferences [58], and we suggest that agent-based simulations can be used to generate highly structured and even observationally biased datasets that allow testing and comparison of these and other network analysis methods.

### (f) Model assumptions

There are several assumptions and caveats to consider when interpreting our results. First, switching rates vary with many factors beyond the individual, including season, location, and roost type. Second, empirical estimates of roost switching were likely influenced by some sampling biases. For instance, roost switching was estimated to be more common among upriver than downriver sites where not all roosts were found [27,39]. Third, when calculating cluster-switching rates and propensities, we assumed that movement between corners of a flight cage was a proxy for movements between locations within a hollow tree or cave. Although this dataset is the best estimate of movement within roosts available, it may differ across roost types. Fourth, there was a higher proportion of males in the empirical roost-switching dataset (wild bats) than in the within-roost datasets (captive bats). If sex was not a key driver of movement rate, then our results remain valid. If, on the other hand, females were less variable in roost switching than males, as might be expected from some empirical observations [27], then these sex ratio differences would cause us to underestimate the extent to which individual variation in between-roost switching is less than in within-roost switching, and this would mean that the effects we detected (the greater impact of within-roost movement) would be greater than what we reported. Researchers should therefore be cautious when generalizing specific findings to new locations or species.

## 5. Conclusion

Social network structure is shaped by social behaviours, but also by nonsocial factors that influence movement, with important implications for relationship formation, pathogen transmission, and information flow [12,60–62]. Real animal movements are caused by multiple factors (e.g. social preferences, spatial preferences, environmental factors and individual traits) that cannot be disentangled in observational studies. For example, an individual might move around and encounter more individuals because it lives in a particular habitat type, has a greater desire for social encounters, has a greater territory size, has more energy or ability to move, is more exploratory, has a greater need to forage, or some combination of these factors. Our agent-based reference model allows us to understand the complexity of the social system by adding or removing effects of interest in a stepwise fashion, revealing insights not possible from observational data.

This study on vampire bat social dynamics illustrates one case of how social network centrality can be impacted in complex and perhaps surprising ways by hierarchically embedded scales of movement, even in the absence of social preferences. Hierarchically embedded scales of movement complicate the causal relationship between movement propensity and social connectedness and create a need to identify the proper scale of observation when creating social networks.

## Data Availability

Data are accessible via Dryad [63]. NetLogo and R code is accessible via Zenodo [64]. Supplementary material is available online [65].
